# Supplementary material for: Functional culture and in vitro genetic and small-molecule manipulation of adult mouse cardiomyocytes
Source: Commun Biol. 2020 May 11;3:229. doi: 10.1038/s42003-020-0946-9 (PMC7214405; doi:10.1038/s42003-020-0946-9)
Supplement: Supplementary file 1 — Supplementary Information [file 42003_2020_946_MOESM1_ESM.pdf]

Functional culture and *in vitro* manipulation of adult mouse cardiomyocytes

Supplementary Information

Contents:

Supplementary Figure 1. Uncropped immunoblots for a) Phospho (S473) Akt to Akt ratio normalized to α-tubulin, b) FLAG-PLN normalized to α-tubulin, and c) Reep5 normalized to α-tubulin.

Supplementary Figure 2. Sample TFM workflow demonstrating gel fabrication, stack acquisition, and data analysis. a) Preparation of a widefield-compatible TFM gel using 500 nm fluorescent microbeads. b) A strain field was constructed from frames of relaxation and maximal contraction before translation to a stress field using the Young's modulus and Poisson's ratio of the polyacrylamide gel. The resulting uniaxial stress vectors were integrated within the projected area of the cell and divided by 2 to obtain a whole-cell net traction force.

Supplementary Table 1.

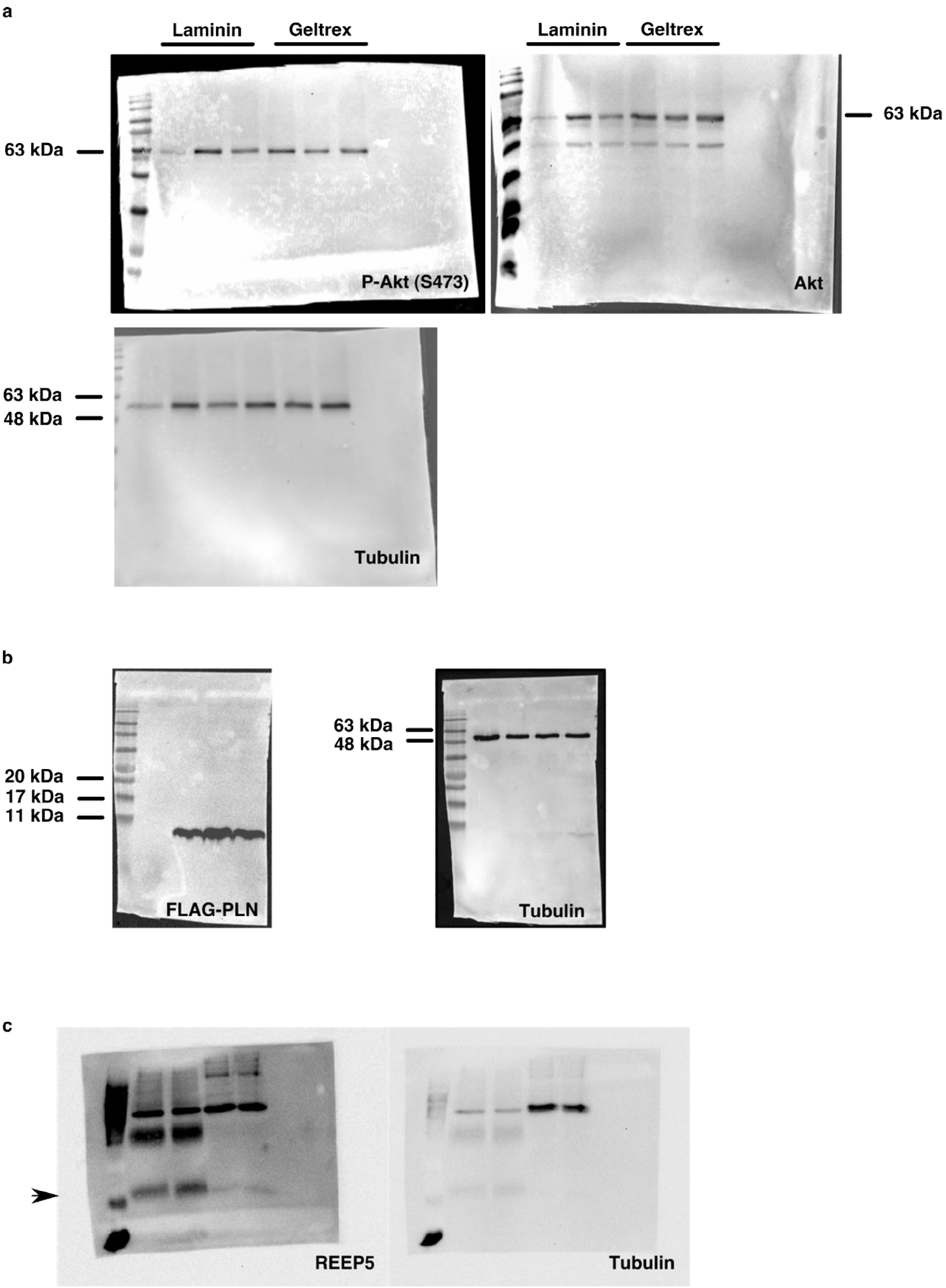

Supplementary Figure 1.

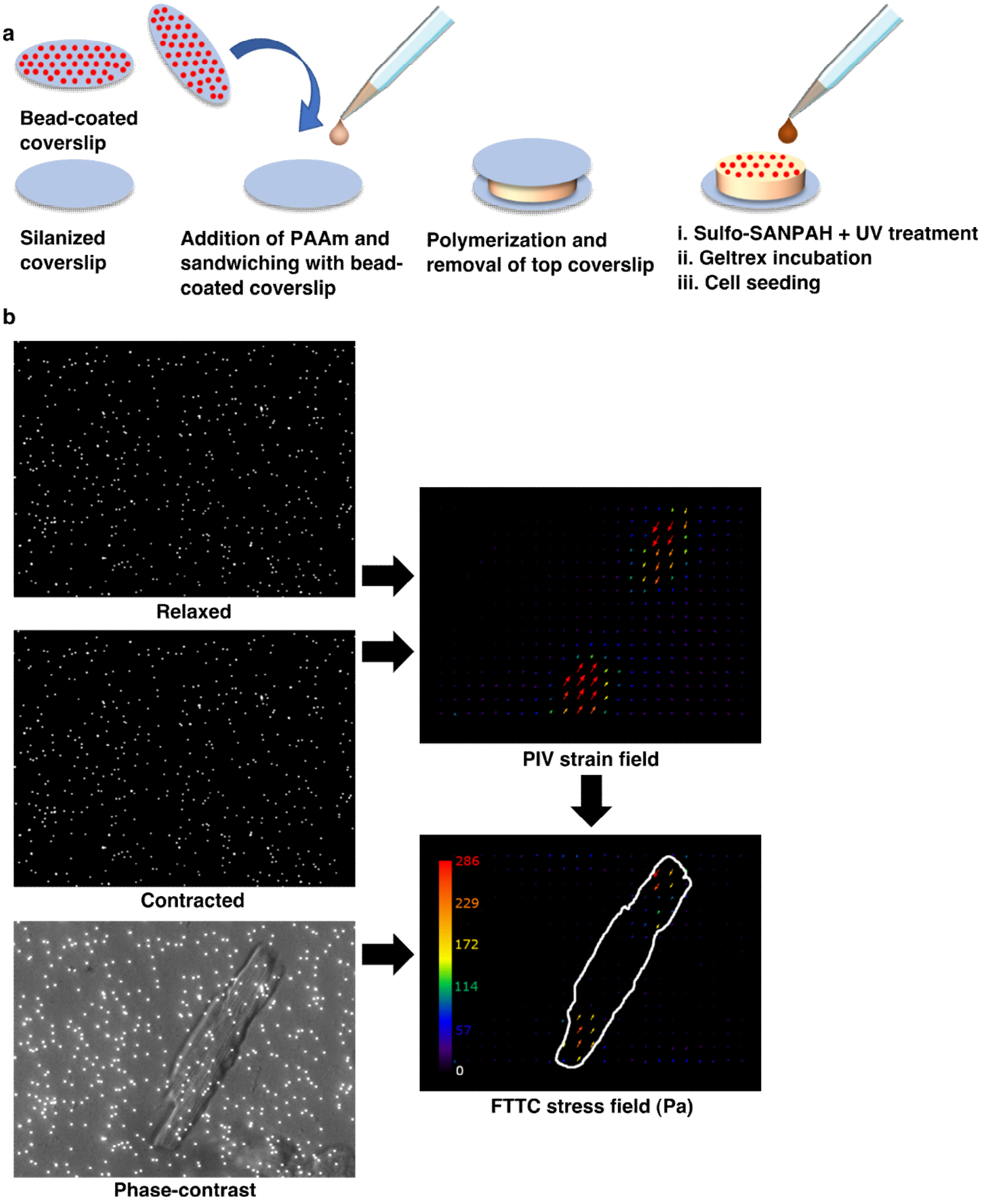

Supplementary Figure 2.

Supplementary Table 1. Composition of EDTA buffer, perfusion buffer, cell culture medium, and plating medium. All buffers are made in >18 MΩ deionized water, sterile-filtered, and replaced frequently.

Buffer	Molar mass (g mol^{-1}) or stock concentration	Final concentration	Amount per 100 mL
**EDTA buffer (pH 7.80)**			
NaCl	58.44	130 mmol L^{-1}	759.7 mg
KCl	74.55	5 mmol L^{-1}	37.3 mg
NaH_2PO_4	119.98	0.5 mmol L^{-1}	6.0 mg
HEPES	238.3	10 mmol L^{-1}	238.3 mg
Glucose	180.16	10 mmol L^{-1}	180.2 mg
Taurine	125.15	10 mmol L^{-1}	125.2 mg
EDTA	292.24	5 mmol L^{-1}	146.1 mg
Blebbistatin*	292.33	0.015 mmol L^{-1}	438 µg
**Perfusion Buffer (pH 7.80)**			
NaCl	58.44	130 mmol L^{-1}	759.7 mg
KCl	74.55	5 mmol L^{-1}	37.3 mg
NaH_2PO_4	119.98	0.5 mmol L^{-1}	6.0 mg
HEPES	238.3	10 mmol L^{-1}	238.3 mg
Glucose	180.16	10 mmol L^{-1}	180.2 mg
Taurine	125.15	10 mmol L^{-1}	125.2 mg
$MgCl_2$	95.21	1 mmol L^{-1}	9.5 mg
Blebbistatin*	292.33	0.015 mmol L^{-1}	438 µg
Collagenase type II†	-	475 U mL^{-1}	47500 U
FBS‡	-	10%	10 mL
**Culture medium (pH 7.60)****			
M199 (with $NaHCO_3$, HEPES, L-glutamine, and 5 mM glucose)	-	-	97 mL
Chemically defined lipid supplement	100X	1X	1 mL
Insulin-transferrin-selenium supplement	100X	1X	1 mL
Penicillin/Streptomycin	100X	1X	1 mL
Blebbistatin*	292.33	0.015 mmol L^{-1}	438 µg
FBS‡‡	-	5%	5 mL

*Blebbistatin should be added fresh daily from a dry or DMSO frozen stock. Do not expose to light.

†Add collagenase type II only to create fresh collagenase buffer.

‡Add FBS without collagenase to create stop buffer. Make fresh daily.

**Bicarbonate-free, blebbistatin-free cell culture medium can be used for contractile analysis. Cells should begin beating <5 min after medium change.

‡‡Add FBS to create plating medium. Adjust other volumes accordingly. Make fresh daily.
