# Supplementary material for: Functional culture and in vitro genetic and small-molecule manipulation of adult mouse cardiomyocytes
Source: Commun Biol. 2020 May 11;3:229. doi: 10.1038/s42003-020-0946-9 (PMC7214405; doi:10.1038/s42003-020-0946-9)
Supplement: Supplementary file 2 — Description of Additional Supplementary Files [file 42003_2020_946_MOESM2_ESM.pdf]

Description of Additional Supplementary Files

File Name: **Supplementary Data 1**
Description: Source file containing data for all graphs.
