# Supplementary material for: Functional culture and in vitro genetic and small-molecule manipulation of adult mouse cardiomyocytes
Source: Commun Biol. 2020 May 11;3:229. doi: 10.1038/s42003-020-0946-9 (PMC7214405; doi:10.1038/s42003-020-0946-9)
Supplement: Supplementary file 4 — Reporting Summary [file 42003_2020_946_MOESM4_ESM.pdf]

nature research

Corresponding author(s): Anthony Gramolini

Last updated by author(s): Apr 3, 2020

Reporting Summary

Nature Research wishes to improve the reproducibility of the work that we publish. This form provides structure for consistency and transparency in reporting. For further information on Nature Research policies, see Authors & Referees and the Editorial Policy Checklist.

Statistics

For all statistical analyses, confirm that the following items are present in the figure legend, table legend, main text, or Methods section.

n/a	Confirmed	
☐	☒	The exact sample size (*n*) for each experimental group/condition, given as a discrete number and unit of measurement
☐	☒	A statement on whether measurements were taken from distinct samples or whether the same sample was measured repeatedly
☐	☒	The statistical test(s) used AND whether they are one- or two-sided<br>*Only common tests should be described solely by name; describe more complex techniques in the Methods section.*
☒	☐	A description of all covariates tested
☐	☒	A description of any assumptions or corrections, such as tests of normality and adjustment for multiple comparisons
☐	☒	A full description of the statistical parameters including central tendency (e.g. means) or other basic estimates (e.g. regression coefficient) AND variation (e.g. standard deviation) or associated estimates of uncertainty (e.g. confidence intervals)
☐	☒	For null hypothesis testing, the test statistic (e.g. *F*, *t*, *r*) with confidence intervals, effect sizes, degrees of freedom and *P* value noted<br>*Give P values as exact values whenever suitable.*
☒	☐	For Bayesian analysis, information on the choice of priors and Markov chain Monte Carlo settings
☒	☐	For hierarchical and complex designs, identification of the appropriate level for tests and full reporting of outcomes
☒	☐	Estimates of effect sizes (e.g. Cohen's *d*, Pearson's *r*), indicating how they were calculated

Our web collection on statistics for biologists contains articles on many of the points above.

Software and code

Policy information about availability of computer code

Data collection	MicroManager (v1.4, Vale Lab), Wave (v2.6.0, Agilent), Zen (v2.3, Zeiss)
Data analysis	ImageJ (v1.51j8, NIH), ThunderSTORM plugin for ImageJ (v1.3, Hagen Lab), PIV and FTTC plugins for ImageJ (Théry Lab), Prism (v5, Graphpad Software Inc.), JMP (v11, SAS Institute Inc.), Matlab (R2018a, MathWorks)

For manuscripts utilizing custom algorithms or software that are central to the research but not yet described in published literature, software must be made available to editors/reviewers. We strongly encourage code deposition in a community repository (e.g. GitHub). See the Nature Research guidelines for submitting code & software for further information.

Data

Policy information about availability of data

All manuscripts must include a data availability statement. This statement should provide the following information, where applicable:
- Accession codes, unique identifiers, or web links for publicly available datasets
- A list of figures that have associated raw data
- A description of any restrictions on data availability

All data is available upon reasonable request to the corresponding author.

Field-specific reporting

Please select the one below that is the best fit for your research. If you are not sure, read the appropriate sections before making your selection.

☒ Life sciences ☐ Behavioural & social sciences ☐ Ecological, evolutionary & environmental sciences

Life sciences study design

All studies must disclose on these points even when the disclosure is negative.

Sample size	All treatments or analyses were performed in 3 to 6 separate experiments, except for individual cell forces that were measured for 20 cells on different substrates to illustrate population distribution. Sample sizes for each experiment are specified in the text.
Data exclusions	No data were excluded from the manuscript.
Replication	Treatment-specific effects were independently replicated >3 times for each experiment, using different batches or chemical vectors each time.No reporter genes were used in transduced cells, so measurements were collected agnostic as to the transduced state of the cell.
Randomization	Treatment-specific samples were measured in a random order where appropriate.
Blinding	Researchers were not blinded to the animals used, as all metrics were quantitative.

Reporting for specific materials, systems and methods

We require information from authors about some types of materials, experimental systems and methods used in many studies. Here, indicate whether each material, system or method listed is relevant to your study. If you are not sure if a list item applies to your research, read the appropriate section before selecting a response.

Materials & experimental systems

n/a	Involved in the study	
☐	☒	Antibodies
☒	☐	Eukaryotic cell lines
☒	☐	Palaeontology
☐	☒	Animals and other organisms
☒	☐	Human research participants
☒	☐	Clinical data

Methods

n/a	Involved in the study	
☒	☐	ChIP-seq
☒	☐	Flow cytometry
☒	☐	MRI-based neuroimaging

Antibodies

Antibodies used	All antibodies, concentrations, and product numbers used are provided in the Methods section of the manuscript.
Validation	All antibodies used were previously validated on mice; citations for use were given.

Animals and other organisms

Policy information about studies involving animals; ARRIVE guidelines recommended for reporting animal research

Laboratory animals	Male CD1 or PLNKO mice of 8 weeks or older were used in this study.
Wild animals	N/A
Field-collected samples	N/A
Ethics oversight	Ethics oversight was provided by the University of Toronto Animal Care Committee.

Note that full information on the approval of the study protocol must also be provided in the manuscript.
